# The Invasive Weed *Trianthema portulacastrum* in Israel

**DOI:** 10.3390/plants13040518

**Published:** 2024-02-14

**Authors:** Yaakov Goldwasser, Onn Rabinowitz, Guy Achdary, Omer Kapiluto, Jackline Abu-Nasser, Evgeny Smirnov, Hanan Eizenberg

**Affiliations:** 1 Valley Farmers Center Ltd., Migdal Haemek 2310001, Israel; 2 Northern Research and Development, Kiryat Shmona 1101600, Israel; onnrab@gmail.com; 3 Newe Ya’ar Research Center, Ramat Yishay 3009500, Israel; achdarig@volcani.agri.gov.il (G.A.); omerki4@gmail.com (O.K.); jackline@volcani.agri.gov.il (J.A.-N.); evegenysm30@gmail.com (E.S.); eizenber@volcani.agri.gov.il (H.E.)

**Keywords:** desert horse purslane, invasive plants, seed germination model, *Trianthema portulacastrum*

## Abstract

*Trianthema portulacastrum* L. (Aizoaceae), commonly known as desert horse purslane or black pigweed, is a C4 dicot succulent annual herb that is widespread in Southeast Asia, tropical America, Africa, and Australia. In Israel, it is an invasive weed of increasing importance in agricultural fields. The aim of this study was to investigate the biology of this invasive weed and its spread in the Hula Valley of Israel. Initial studies included the investigation of the *T. portulacastrum* specimens held at the Israel National Herbarium. On-site surveillance for the identification of weed infestation locations was conducted in the Hula Valley throughout 2019–2022, and an infestation map was assembled. In a study of the plant biology, greenhouse pot experiments revealed that *T. portulacastrum* seeds emerge best from the upper soil levels, and as seed depth increases, the emergence rate decreases, so that at 6 cm soil depth, there was no emergence. In controlled-environment growth chamber studies, there were no significant differences in germination with or without light. A maximum germination of 81% was observed for a 12 h night/day of 25/35 °C regime. Germination rates decreased with the decrease in temperature. A seed germination thermal time model that was developed for estimating the minimum temperature required for germination (T_base_) computed this temperature to be 10 °C. This study revealed the biology, in particular seed germination and emergence requirements, of the invasive weed *T. portulacastrum* that has spread in the Hula Valley in Israel and beyond. Future research will focus on an examination of control measures to combat this invasive weed.

## 1. Introduction

**Figure 1:**
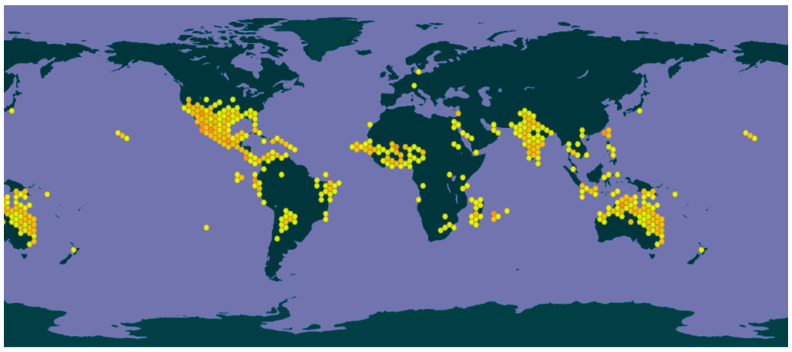
Global distribution of *T. portulacastrum* according to the Global Biodiversity Information Facility (GBIF) (www.gbif.org/species/3084848). Accessed on 15 December 2023.

*Trianthema portulacastrum* L. (Aizoaceae), commonly known as desert horse purslane or black pigweed, is a cosmopolitan plant, being widespread in Southeast Asia, America, Africa, Australia, and Asia, with an unknown center of origin [1] (Figure 1). It is an annual C4 dicot branched, prostrate succulent herb, with spongy, obovate, simple leaves and pink bisexual five-petal flowers that are insect-pollinated. The flowers produce capsules that burst open to spread numerous small, hard-coat seeds throughout the spring, summer, and autumn. The seeds are disseminated via soil and water and remain viable in soil for many years [1,2].

*Trianthema portulacastrum* is a prevalent, easily visible weed affecting a variety of agricultural and vegetable crops in different parts of the world, including pigeon pea, mung bean, cotton, pearl millet, sugarcane, oilseeds, onion, potato, maize, mustard, direct-seeded rice, and tomato [1,3,4,5,6,7]. Although *T. portulacastrum* is generally considered a noxious weed, different plant tissues are used medicinally for their antimicrobial, analgesic, anti-inflammatory, anti-hyperglycemic, and hepato-protective properties [6,8,9].

There have been numerous reports on this weed as an invasive species in the Middle East and in the Indian subcontinent. For example, in Egypt, *T. portulacastrum* has been reported to be a troublesome invasive weed in orchards and in maize, cotton, and soybean fields, with high phenotypic plasticity due to different environmental conditions of soil moisture, soil salinity, and temperature regimes in the various geographical growing regions [10]. Controlled-environment laboratory seed germination studies of 35 Egyptian *T. portulacastrum* populations revealed that a single individual plant produces an average of 1931 seeds, with a seed average dry weight of 1.08 g, and that high temperatures of 30–45 °C promote maximum seed germination [11]. Reports from India and Pakistan also describe this plant as an invasive and pernicious weed in many fields having only limited control measures. A study of chemical and cultural control measures showed that, to accomplish efficient weed control, it was necessary to apply these measures in the early growth stages—up to 40 days after emergence [4,5]. In other studies, cultural and biological measures for *T. portulacastrum* control also achieved only limited success [12,13,14] (Aneja, 2010; Khalik et al., 2011; Saeed et al., 2010). 

Recent reports of invasions into Jordan and Israel led, in 2019, to the inclusion of *T. portulacastrum* on the invasive weed alert list of the European and Mediterranean Plant Protection Organization (EPPO), an intergovernmental organization responsible for cooperation on plant health within the Euro-Mediterranean region [15]. Accessed on 9 November 2023). In Israel, the exact locations and extent of the weed invasions have not been studied—with the exception of the current study pertaining to the Hula Valley of northern Israel.

The Hula valley is located at the north-east tip of Israel (Figure 1) spreading throughout 200 km^2^, at an average elevation of 70 m above sea level. The climate of the Hula valley is Mediterranean, with hot dry summers and cool rainy winters. Annual rainfall ranges from 400 mm in the south of the valley, to up to 800 mm in the northern part of the valley. The mountain-enclosed topography of the valley leads to more extreme daily and seasonal temperature fluctuations, compared to the adjacent coastal climate. The center of the valley was swamp-dried in the 1950s, and since then has developed into a center of modern intensive agriculture consisting of orchards, field crops, and vegetable farming.

*Trianthema portulacastrum* establishment in the valley is not surprising, since this invasive weed thrives in warm, moist soil environments and easily acclimates to the agricultural conditions of the irrigated summer crops in the valley. Recent reports of this weed in vegetable and field crops and waterway edges in the Hula Valley have emphasized the need to conduct research on the degree of invasiveness of this weed and on its biological traits, as a baseline for establishing efficient general and crop-selective control measures. 

The aim of this study was therefore to detect and map *T. portulacastrum* invasions in the Hula Valley and to study the biology and environmental conditions that expedite its fast invasiveness, including the development of a thermal time seed-germination model. 

## 2. Materials and Methods

### 2.1. Trianthema portulacastrum at the Israel National Herbarium

The current investigation started with an examination of the dried *T. portulacastrum* specimens held in the Israel National Herbarium (https://nnhc.huji.ac.il/herbarium/?page_id=29&lang=en. Accessed on 15 December 2023). The Herbarium, currently located at The Hebrew University of Jerusalem, houses the most comprehensive plant collection in the Middle East—more than one million specimens of dried plants, mainly from Israel but also from neighboring countries—and as such, is a comprehensive information resource for Israeli and international researchers on plant systematics, taxonomy, conservation biology, ecology and biogeography. For this study, all the *T. portulacastrum* plants in the Herbarium were examined and photographed, and the details of their collection were recorded, including the identification of the collector, the collection location, and the date of collection.

### 2.2. Surveillance, Mapping and Plant Biology

During 2019–2022, we conducted ground surveillance of the Hula Valley fields to identify *T. portulacastrum* infestations. Identification of the infestation locations was followed by determination of geographical GPS latitude and longitude coordinates mapping, and the visiting of these sites to study the plant biology and life cycle throughout the crop growing seasons. 

### 2.3. Hula Valley Plant Material

**Figure 2:**
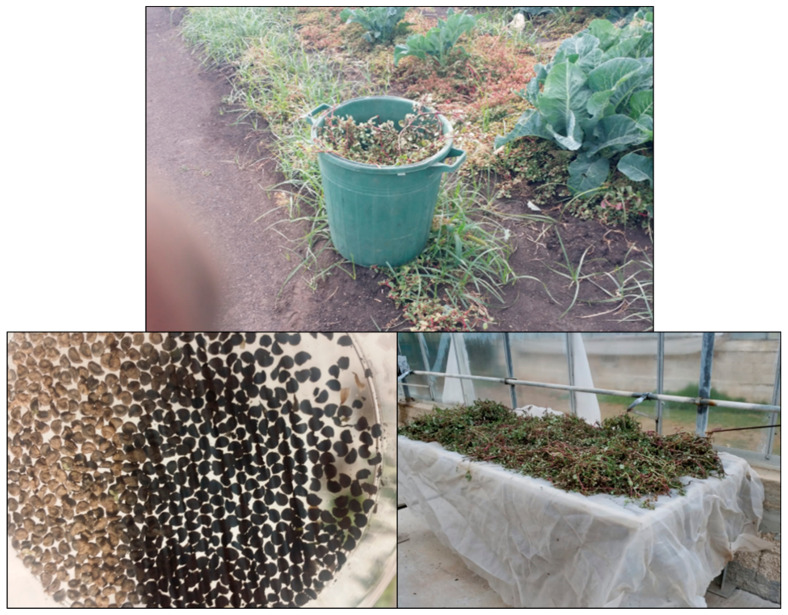
Field collection, greenhouse drying, and final cleaning of *T. portulacastrum* seeds (clockwise from top).

During the study period (2019–2021), mature *T. portulacastrum* plants were collected from three main infestation sites in the Hula Valley. The plants were dried during the summer in the greenhouse, after which they were thrashed, allowing the seeds to fall. The seeds were collected and stored at 4 °C (Figure 2). These seeds were subsequently used for all laboratory, greenhouse, and net-house germination and emergence experiments.

### 2.4. Germination Experiments

***Preliminary studies***. A series of preliminary experiments was conducted to determine the efficient conditions facilitating *T. portulacastrum* germination. Initially, we obtained very low germination rates for non-treated seeds germinated on water-moistened fiber paper in Petri dishes under a wide range of temperatures. Further experimentation with different seed treatments and growth promoters or substrates resulted in a high seed germination treatment: seeds were immersed in 97% sulfuric acid for 30 min, thoroughly washed with sterile water, and seeded in covered Petri dishes or plates filled with sterilized 70% Murashige and Skoog (MS) agar. All procedures were performed under a sterilized laminar flow hood.

**Figure 3:**
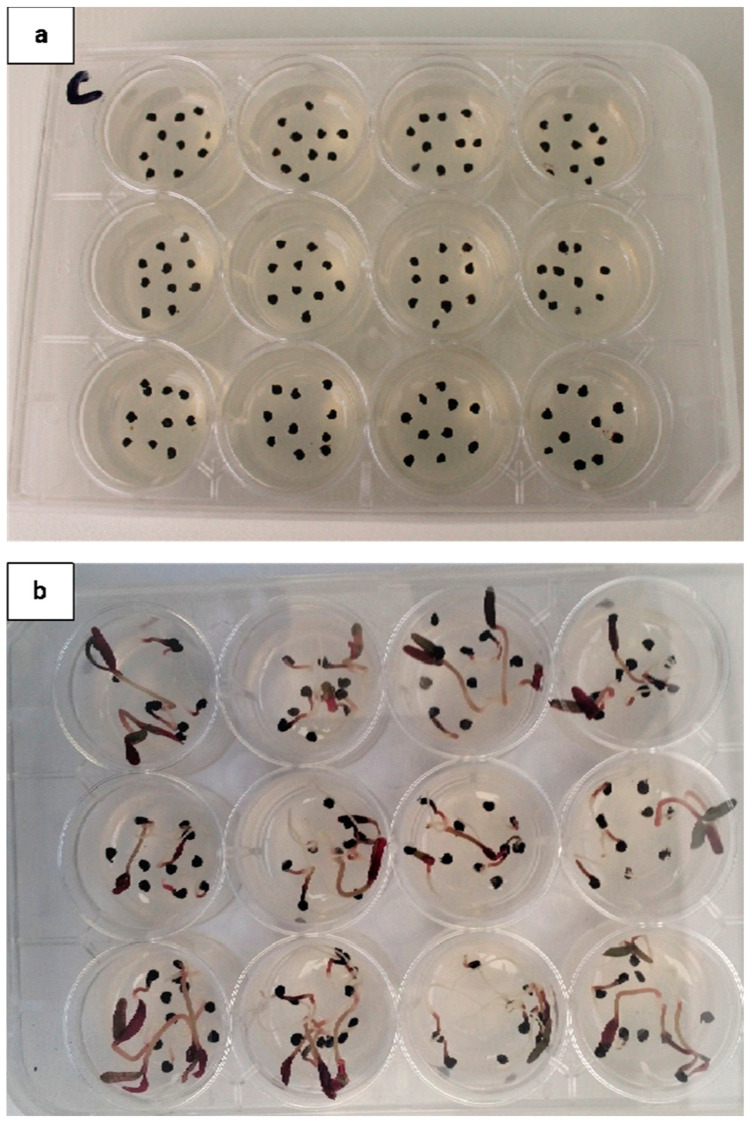
*Trianthema portulacastrum* in a 12-well plate in a temperature-/light-controlled environment germination assay. (**a**) Seed placement on the day of seeding; 10 seeds per well. (**b**) Germination of *T. portulacastrum* in the 25/35 °C dark treatment 13 days after seeding.

***Temperature-light-controlled environment germination assay***. Following the preliminary tests, all germination experiments were conducted in 12-well cell culture plates filled with 3 mL of 70% MS agar; the plates were seeded with 10 seeds per well (Figure 3). The experiments were conducted in climate-controlled LED-lit plant growth chambers (model ST B SMART, ‘Conviron’) preset to alternating temperatures of 10/20, 15/25, 20/30, or 25/35 °C either in the ‘light’ (a 12 h dark/12 h light photoperiod) or in ‘dark’ (complete darkness achieved by wrapping the 12-well cell culture plates with aluminum foil). All treatments were conducted with 12 replications (namely, 12 wells) per treatment in a completely randomized design. *T. portulacastrum* seedling germination rates were recorded daily, and the experiment was terminated 17 days after seeding (DAS).

***Seedling emergence experiments***. *Trianthema portulacastrum* plant emergence experiments were conducted in 800 mL plastic pots filled with Newe Ya’ar soil (57% clay, 22% silt, 21% sand, 2% organic matter, pH 7.7). Ten *T. portulacastrum* seeds per pot were seeded at depths of 0 to 6 cm in 1 cm increments, 6 replications per depth. Following seeding, the pots were placed in a net-house and watered by mini-sprinklers as needed. Plant emergence was recorded weekly, and the experiment was terminated 22 DAS.

***Development of a seed germination model***. There were no significant differences between seed germination rates in light and dark conditions under all the examined temperature regimes, and therefore, data from light and dark conditions were pooled. Observations for each 12-well cell culture plate and temperature regime were used to parameterize a time-to-event model [16]. The association between the cumulative seed germination and time (in days) under the four different temperature regimes were analyzed using a log-logistic equation:

(1) $$Y(t)=d/(1+exp\left(b\left(log\left(t\right)-log\left(e\right)\right)\right)$$

where *Y* is the proportion of germinated seeds at time *t*, *d* is the proportion of maximum germinated seeds when *t → ∞*, *e* is the median germination time, and *b* is the steepness of the curve at the inflection point. 

A thermal time germination model for *T. portulacastrum* was fitted to the four-parameter Weibull equation by using nonlinear regression:

(2) $$f\left(x\right)=a\times \left[1-\exp\left(-{\left(\frac{GDD-lag}{b}\right)}^{c}\right)\right]$$

where *a* is the maximal field *T. portulacastrum* germination, *c* is the shape parameter that determines the skewness and kurtosis of the equation, and *b* is a scale parameter regardless of the shape value when germination rate is 63%.

All statistical analyses were performed using the R statistical environment (R Core Team 2020) together with the following packages: ‘drc’ [17] and ‘drcSeedGerm’ [18].

## 3. Results

### 3.1. Trianthema portulacastrum at the Israel National Herbarium

**Figure 4:**
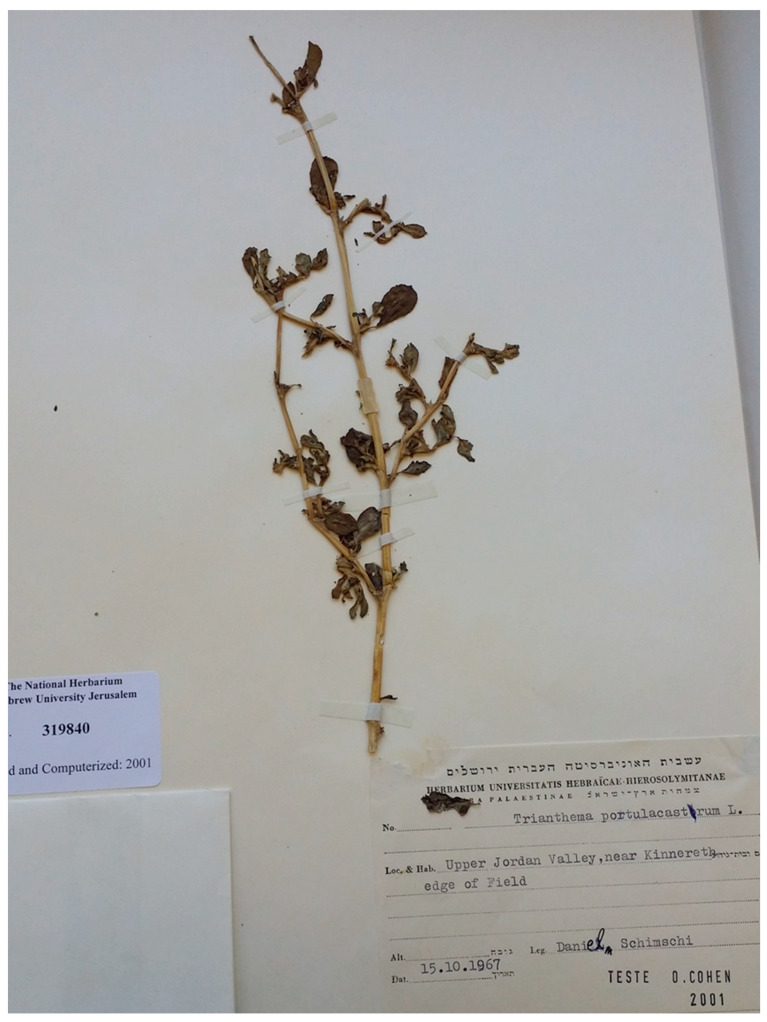
The first *T. portulacastrum* sample in the Israel National Herbarium, collected by Daniel Schimshi in 1967 at the edge of a field near Kibbutz Kinneret in the Upper Jordan Valley.

The *T. portulacastrum* specimens stored in the Israel National Herbarium were retrieved and photographed, and the collection details were recorded and summarized (Table 1). The first specimen was collected in 1967 from a field edge near Kibbutz Kinneret at the south-west edge of the Sea of Galilee (Figure 4). Specimens collected in the 1970s and 1980s were obtained from areas across the breadth of the country, namely, fields in the region south of Lake Kinneret (the Jordan Valley and the Beit Shaan Valley), fields at Glil Yam and Ma’agan Michael located in the central coastal plain, and a roadside near Ein HaHoresh in the Hefer Valley adjacent to the central coastal plain. In the 1990s and onwards, samples were collected from the Hula Valley (in 1995 from Lehavot HaBashan and 2010 from Agamon Hahula), the Jordan Valley, and the central coastal plain. Overall, the *T. portulacastrum* plant specimens were collected from a wide range of sites—fields, orchards, canal ditches, roadsides, and riversides.

### 3.2. Trianthema portulacastrum Survey, Mapping and Plant Biology in the Hula Valley

**Figure 5:**
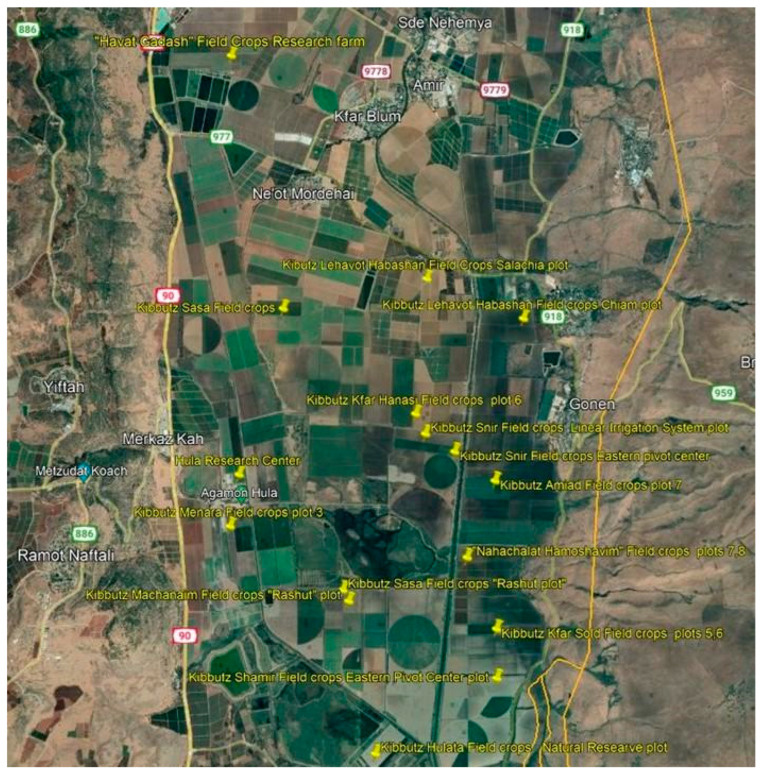
*Trianthema portulacastrum* distribution map in the Hula Valley, as recorded for 2019–2022.

**Figure 6:**
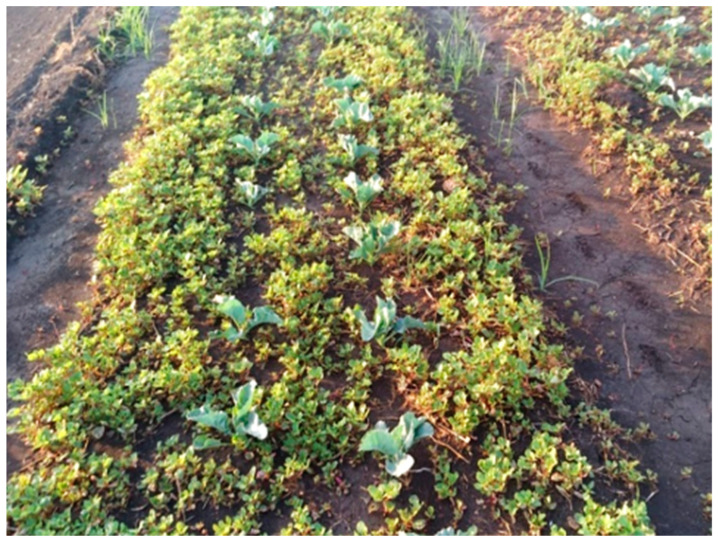
Heavy *T. portulacastrum infestation* in a broccoli field, Manara, Hula Valley, October 2020.

**Figure 7:**
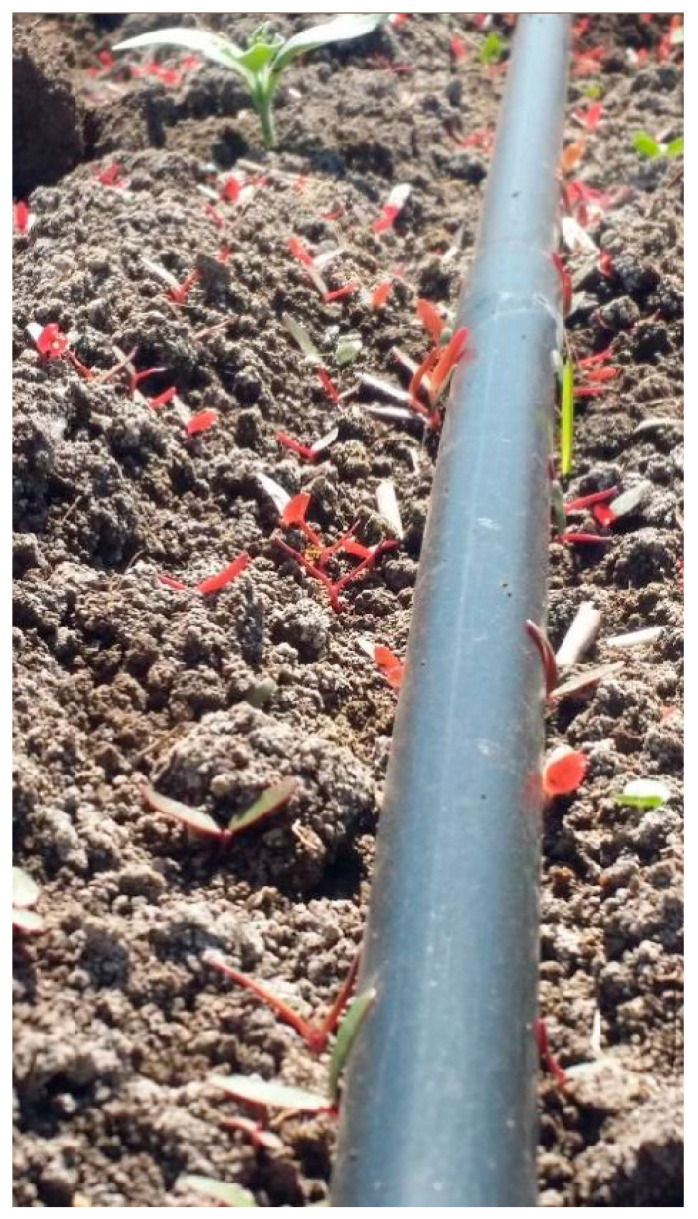
*Trianthema portulacastrum* seedlings emerging adjacent to a drip irrigation line in a processing tomato field, Lehavot Habashan, Hula Valley, June 2021.

**Figure 8:**
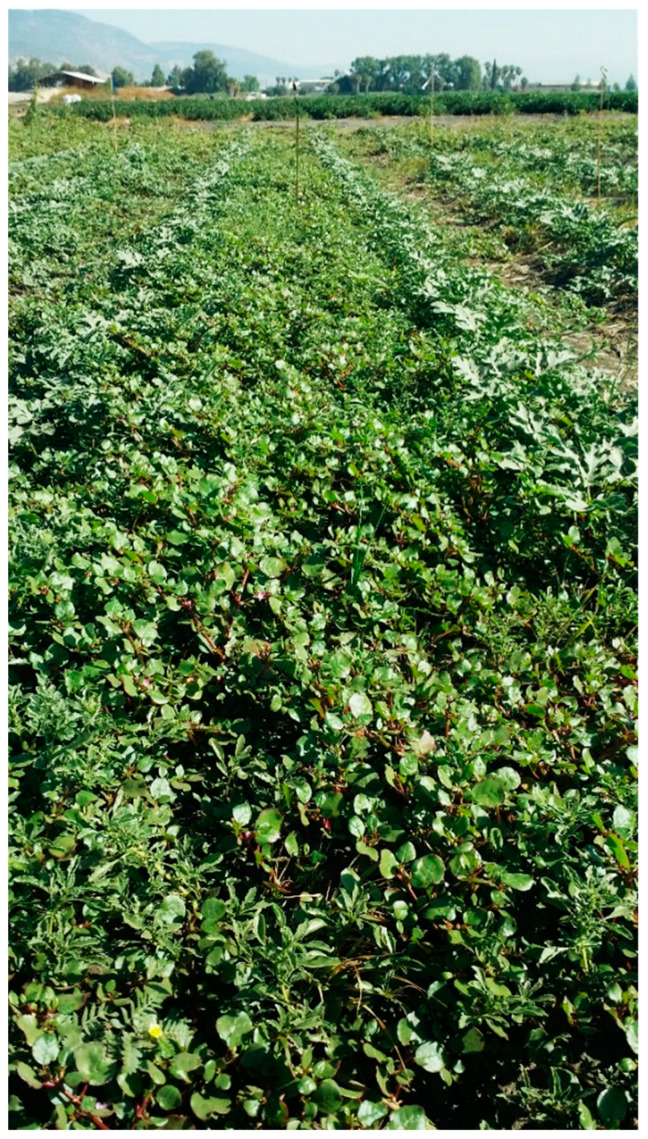
*Trienthema portulacastrum* heavy infestation in a watermelon field, Manara, Hula Valley, June 2021.

**Figure 9:**
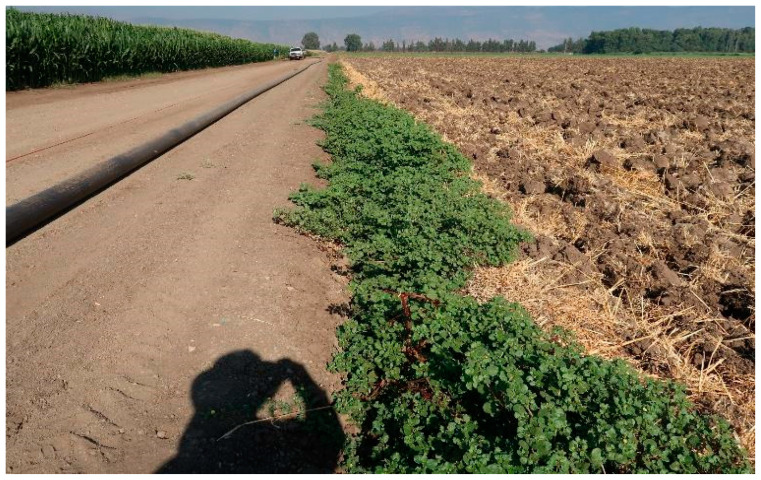
*Trianthema portulacastrum* infestation along a field margin, Lehavot Habashan, Hula Valley, August 2019.

A survey of the Hula Valley region identified 16 main sites infested with *T. portulacastrum* (Figure 5). The invasive weed was widespread in field and vegetable crops (maize, teff, groundnuts, setaria, watermelon, tomato, potato, and broccoli), and there were also heavy infestations of field boundaries, waterways banks, and other moist areas (Figure 6, Figure 7, Figure 8 and Figure 9). The weed started emerging in early April, flowering 3–4 weeks later, and produced seeds throughout the summer until late autumn.

**Figure 10:**
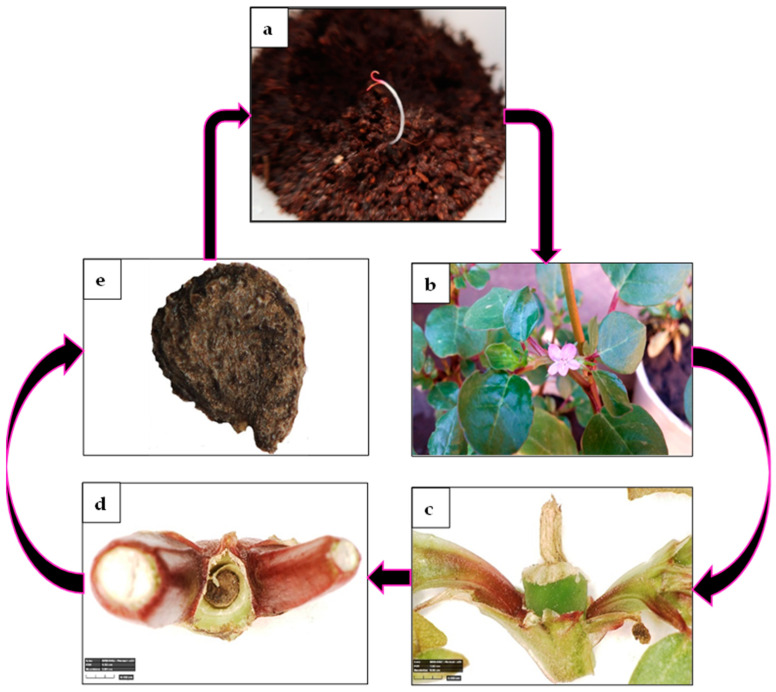
*Trianthema portulacastrum* life cycle as portrayed by photographs from the present study. (**a**) Cotyledon stage seedling. (**b**) Mature flowering plant. (**c**) Capsule. (**d**) Mature open capsule with seeds remaining at the capsule base. (**e**) Mature seed.

A study of the plant biology revealed that the plants have solitary, star-shaped, pink bisexual flowers with a lifespan of one day. The flowers develop in leaf axils, open in the morning, and are insect-pollinated. Small (2 to 3 mm in diameter), kidney-shaped brown to black hard-coat seeds develop in cylindrical capsules embedded in the stems. These dehiscence capsules, each containing 3–8 seeds, mature within days. They open along the operculum, releasing most of the seeds to the ground, while the 1–3 seeds remaining in the capsule base are dispersed with the plant. A photographic depiction of the life cycle derived from our observations and experiments is presented in Figure 10.

### 3.3. Temperature-/Light-Controlled Environment Germination Assay

**Figure 11:**
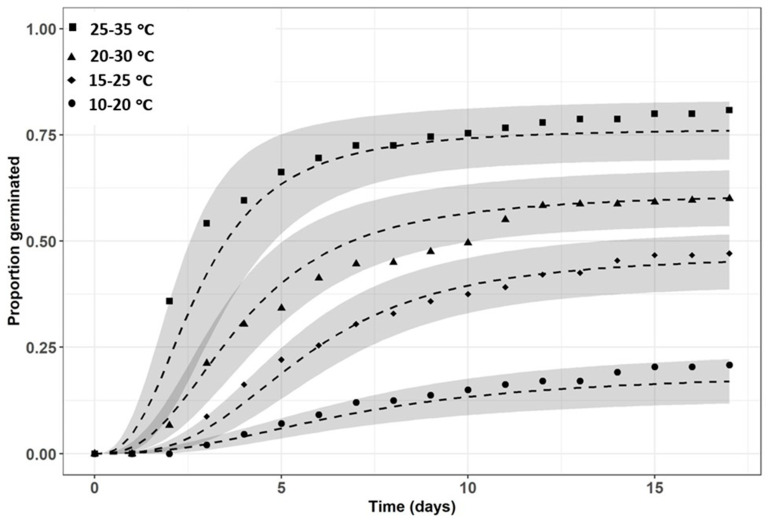
Log-logistic Equation (1) fitted to estimate *T. portulacastrum* seed germination proportions under four temperature regimes. Coefficients for the equation and statistical parameters are presented in Table 2.

The controlled-environment (temperature/light) seed germination tests showed that the *T. portulacastrum* seed germination rate increased with an increase in the temperature regime. Thus, at the highest tested temperature regime of night/day 25/35 °C, a very high germination rate of 81% was recorded at the end of the experiment (17 DAS), compared to only 23% seed germination at the lowest 10/20 °C temperature regime tested (Figure 11; Table 2, parameter d). Germination of *T. portulacastrum* seeds in the dark vs. in the light yielded results that were not statistically different for all the temperature regimes, and therefore data from the light and dark treatments were combined (Figure 11).

**Figure 12:**
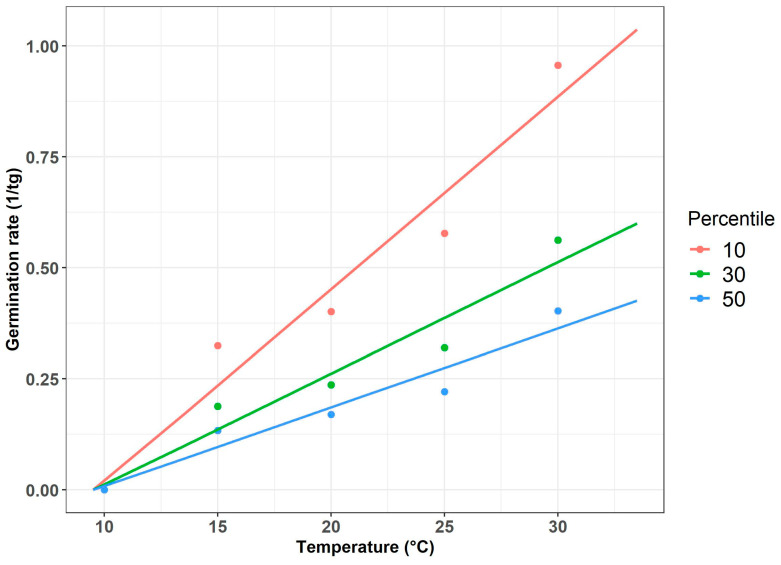
*T. portulacastrum* seed germination rate at three percentiles (10, 30, 50).

*Trianthema portulacastrum* seed germination rate at three percentiles (10, 30, 50) was computed for estimating the minimal temperature (T_base_) that is required for germination; this temperature was found to be 10 °C (Figure 12). 

**Figure 13:**
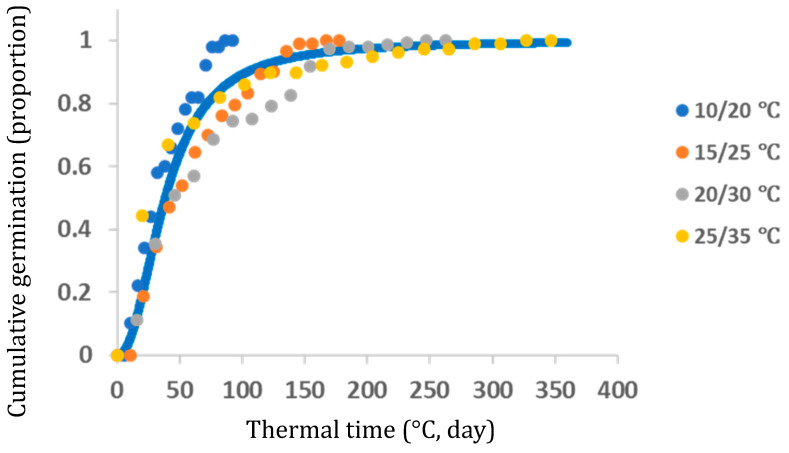
The thermal time (°C days) model for estimating cumulative germination proportion of normalized data based on four temperature regimes (% of the maximal germination).

A thermal time (in °C days) model for estimating cumulative germination proportion of normalized data based on four temperature regimes (% of the maximal germination) was computed. The model estimations were as follows: the upper asymptote (parameter a) 0.97; the scale parameter 40.05, meaning 63% germination; the shape parameter 1.53; and the lag phase to the first germination 9.12 °C days (Figure 13).

### 3.4. Seedling Emergence Test

**Figure 14:**
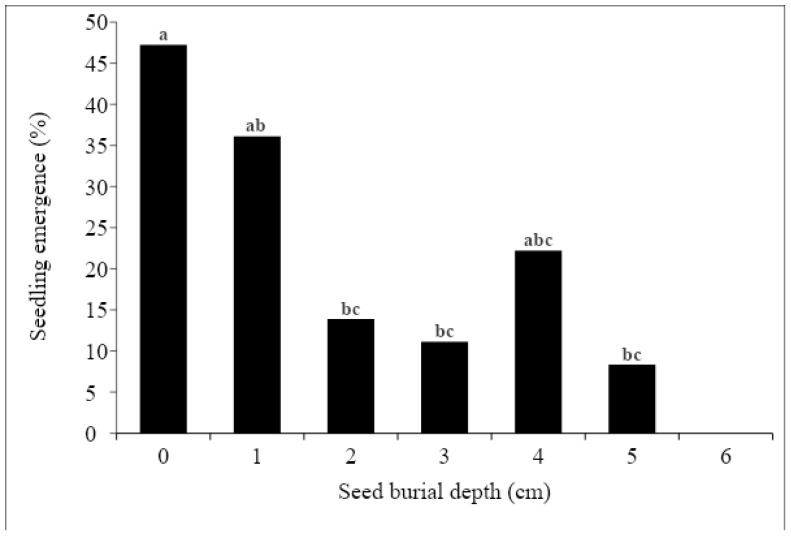
Emergence percent 22 DAS for *T. portulacastrum* seedlings for different soil depths in the greenhouse pot experiment. Emergence rates with different letters are statistically different according to the Tukey–Kramer multiple range test, α = 0.05.

In the experiment conducted in a greenhouse with *T. portulacastrum* seeded in pots, we found that cotyledons started emerging 12 DAS from the 0–1 cm soil depth. The maximum germination rate was achieved for the 1 cm-depth treatment, i.e., 36% germination at 22 DAS. The initial germination of seeds that were placed on top of the soil (0 cm) was slower, but increased with time, reaching 47.2% germination at the end of the experiment (22 DAS). As seed placement depth increased, germination rates dropped, so that at the 6 cm seeding depth, plants did not emerge at all Figure 14.

## 4. Discussion

In this study, we elucidated the spread and biology of the invasive weed *T. portulacastrum* in the Hula Valley of Israel. This troublesome weed is well-established in the agricultural regions of the Hula Valley, and infestation reports reveal that it is spreading rapidly from the Hula Valley in the north to the Arava Valley in the south of Israel. The weed thrives in warm weather and moist conditions, as reported in the USA, Central Africa, Australia, India, Sri Lanka, Pakistan, and Egypt. Such climatic conditions are typical for summer-irrigated crops in the Mediterranean climate of Israel, promoting the spread of this invasive species in agricultural ecosystems, including fields, roadsides, and waterway banks. Our current study showed that the hard seed coat is responsible for the mechanical dormancy of the seeds, that germination occurs in the light as well as in dark environments, and that the seed emergence rate is highest in conditions of adequate moisture and shallow soil depths, including the soil surface. In experiments in which seeds were germinated in 12-well plates under different night/day temperature regimes, a maximum germination rate of 81% was obtained for the night/day 25/35 °C regime, with a decrease in germination rates as the temperatures decreased. These findings are in agreement with those of [19], who reported maximal seed germination of *T. portulacastrum* in the Philippines at high temperatures of 25/35 °C night/day, and of [11], who reported a maximal seed germination rate of over 95% at 30–45 °C for Egyptian populations, while at 20 °C only 1% germination was observed. In our laboratory studies, *T. portulacastrum* germination rates at light vs. dark regimes did not differ significantly at all temperature regimes tested, in contradiction to the report of [11] of reduced germination in the dark at 25/35 °C. An additional difference between the two studies is that [11] did not mention any required pre-treatment to ensure maximal germination, while in our study, seeds did not germinate without acid pre-treatment.

From the seed germination thermal time model developed in this study, cardinal coefficients were computed; the model showed that the most important parameter that directly affects seed emergence is T_base_, which was computed to be 9.58 ± 1.51 °C (*p* < 0.05). This value is in line with seasonal observations of *T. portulacastrum* emergence under Israeli climatic conditions, where *T. portulacastrum* infests crops that are cultivated in the summer, such as maize, tomato, watermelon, setaria, and groundnut. In a similar thermal time model study on the perennial weed *Solanum elaeagnifolium* in Israel, T_base_ for germination was computed to be 10.8 °C [20]. A seed germination thermal time model was also used by [21], who studied the effects of light, temperature, and soil depth on the germination and emergence of the invasive weed *Conyza canadensis* (L.) Cronq. Their experiments showed that under a 12 h/12 h light/dark photoperiod, the highest germination capacity was obtained at 15 °C; germination rates at 17/23 and 22/28 °C did not differ from those at 20 and 25 °C; and germination was significantly reduced at 12/18 °C. The shortest germination time was obtained at 25 °C (light/dark), and this time increased significantly at 12/18 °C and in the dark. The highest emergence was from a depth of 0 mm. Modeling germination rates as a function of temperature gave T_b_ = 6.8 °C (base temperature) and T_c_ = 35.8 °C (ceiling temperature).

In our pot experiment, *T. portulacastrum* emerged at rates of 47% from the soil surface and 36% from 1 cm depth, and as seeds were placed deeper in the soil, the emergence decreased so that at a 6 cm depth, there was no emergence at all. Similarly, the laboratory studies of [8] in India showed that maximal emergence of *T. portulacastrum* seeds occurred at a 1 cm depth, with the emergence percent declining with an increase in seed depth. However, in contrast to our findings, seeds placed on the soil surface did not emerge at all. 

As mentioned above, the results of a study in which *T. portulacastrum* seeds were collected in corn fields at the Agricultural Research Center of Giza, Egypt, showed that a single plant produces an average of 1931 min seeds, weighing only 1.08 g each [11]. In a different recent study, also conducted in Egypt—covering 35 sites in the Fayoum Depression—[10] concluded that the C4 *T. portulacastrum* plant has a high plasticity index, enabling this invasive plant to adapt, flourish, and spread in different habitats, even those with dry and saline conditions. Our study revealed the production of vast numbers of minute hard-coat seeds, most of which are released to the soil surface. However, some seeds remain in the plant capsule (not detached from plant), with the combination of released/attached seeds ensuring that under natural field conditions the seeds are disseminated over both long and short distances. A high seed germination rate in wet soils throughout the warm periods (April–October) may thus be expected, as was indeed demonstrated in this study. 

This study was conducted in the framework of a national effort to combat and stop the spread of invasive weeds in agriculture in Israel. In recent years, *T. portulacastrum* infestations have increased significantly and are spreading to new regions. Thus, the major importance of this research lies in its investigation of the weed’s prevalence and biology. The data generated will be applied in studies of agro-technical and chemical measures to control and restrict its spread in crops, roadsides, and waterway banks, and to increase the farmers’ and researchers’ awareness of this invasive weed. Currently, we are testing—under controlled conditions and in the field—the efficacy of selective and non-selective *T. portulacastrum* control measures.

Future research will focus on an examination of selective and non-selective herbicide applications under greenhouse and field conditions, aimed to develop tools for efficient *T. portulacastrum* control in crops and along roadsides and waterways.

## Figures and Tables

**Table 1:** Summary of the *T. portulacastrum* specimens in the collection of the Israel National Herbarium.

No.	Year	Settlement	Location	Date	Collector	Herbarium Serial No.
**1**	1967	Kinneret	Field edge	15 October 1967	Schimshi	319840
**2**	1970	Ashdot Yaakov	-	15 September 1970	Pundak	319839
**3**	1979	Glil Yam	Field	2 October 1979	Sando	319845
**4**	1981	Hamadia	Field	24 June 1981	Cohen	319842
**5**	1981	Hamadia	Field	24 June 1981	Danin	319841
**6**	1985	Ma’agan Michael	Field	1 July 1985	Schmida	319846
**7**	1986	Ein Hahoresh	Roadside	4 November 1986	Danin	319848
**8**	1990	Petach Tikva	Riverside	28 October 1990	-	71082
**9**	1995	Lehavot Habashan	-	28 July 1995	Beckerman	319849
**10**	1998	Givat Brenner	Ditchside	4 November 1998	Danin	72538
**11**	1998	Hakfar Hayarok	Citrus orchard	20 August 1998	Fergman	7687
**12**	2001	Beit Zera	Date palm plantation	23 July 2001	Danin	125400
**13**	2008	Agmon Hahula	Field	27 July 2010	Or	-

**Table 2:** Coefficients of log-logistic equations (Figure 11).

Temperature Regime (°C Night/Day)	Coefficient	Estimate	Std. Error	Value	Probability (>|t|)
10/20	b	−2.47505	0.293985	2.20 × 10^−16^	***
	d	0.235825	0.040853	9.25 × 10^−9^	***
	e	7.491596	0.960691	1.08 × 10^−14^	***
15/25	b	−2.55411	0.211338	2.20 × 10^−16^	***
	d	0.502321	0.0311	2.20 × 10^−16^	***
	e	5.892004	0.534219	2.20 × 10^−16^	***
20/30	b	−2.28439	0.168331	2.20 × 10^−16^	***
	d	0.629312	0.038963	2.20 × 10^−16^	***
	e	4.532321	0.44012	2.20 × 10^−16^	***
25/35	b	−2.53927	0.17967	2.20 × 10^−16^	***
	d	0.814441	0.041982	2.20 × 10^−16^	***
	e	2.482942	0.16417	2.20 × 10^−16^	***

d is the proportion of maximum germinated seeds when t → ∞, e is the median germination time, and b is the steepness of the curve at the inflection point. (*** represent P. value <0.001).

## Data Availability

The data is contained within the manuscript.
